# LGR5 and LGR6 in stem cell biology and ovarian cancer

**DOI:** 10.18632/oncotarget.20178

**Published:** 2017-08-11

**Authors:** Adam J. Schindler, Arisa Watanabe, Stephen B. Howell

**Affiliations:** ^1^ Moores Cancer Center, University of California, San Diego, CA, USA

**Keywords:** ovarian cancer, Wnt, LGR6, LGR5, RSPO

## Abstract

Wnt signaling plays a fundamental role in patterning of the embryo and maintenance of stem cells in numerous epithelia. Epithelial stem cells are closeted in niches created by surrounding differentiated cells that express secreted Wnt and R-spondin proteins that influence proliferation rate and fate determination of stem cell daughters. R-spondins act through the LGR receptors to enhance Wnt signaling. This close association of stem cells with more differentiated regulatory cells expressing Wnt-pathway ligands is a feature replicated in all of the epithelial stem cell systems thus far examined. How the stem cell niche operates through these short-range interactions is best understood for the crypts of the gastrointestinal epithelium and skin. Less well understood are the stem cells that function in the ovarian surface epithelium (OSE) and fallopian tube epithelium (FTE). While the cuboidal OSE appears to be made up of a single cell type, the cells of the FTE progress through a life cycle that involves differentiation into ciliated and secretory subtypes that are eventually shed into the lumen in a manner similar to the gastrointestinal epithelium. Available evidence suggests that high grade serous ovarian carcinoma (HGSOC) originates most often from stem cells in the FTE and that Wnt signaling augmented by LGR6 supports tumor development and progression. This review summarizes current information on LGR5 and LGR6 in the OSE and FTE and how their niches are organized relative to that of the gastrointestinal epithelium and skin.

## INTRODUCTION

Wnt proteins activate at least two major intracellular pathways, the canonical and non-canonical Wnt signaling pathways. In the canonical pathway, the binding of Wnt ligands to the cell-surface receptors Frizzled (FZD) and LDL-receptor related protein 5/6 (LRP5/6) leads to the phosphorylation of the LRP receptor and degradation of intracellular GSK3β. This frees β-catenin to migrate to the nucleus and form a complex with TCF/LEF, driving transcription of a large number of genes involved in cell proliferation and stem cell self-renewal [1].

**Figure 1:**
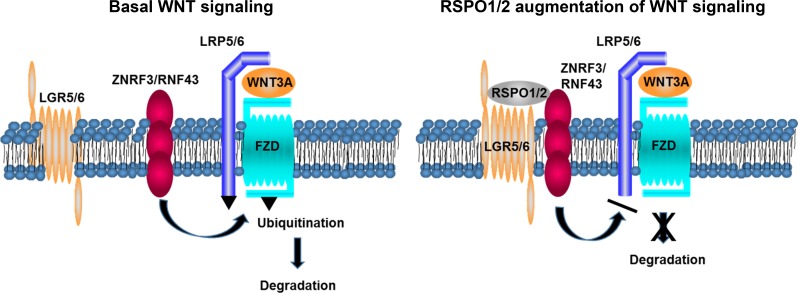
RSPO augmentation of Wnt signaling Left panel: In the absence of RSPO1/2, ubiquitin ligases ZNRF3 and RNF43 ubiquitinate LRP5/6 and FZD receptors, which marks them for lysosomal degradation and limits their residence time on the cell surface. Right panel: Binding of RSPO1 or RSPO2 to LGR5 or LGR6 causes the receptor to clear ZNRF3/RNF43 from the membrane, which prolongs the plasma membrane residence time of LRP5/6 and FZD and augments Wnt signaling.

In addition to Wnts, a second type of ligand secreted from the niche, the R-spondin (RSPO), also regulates Wnt signaling in stem cells. The four isoforms of RSPO (RSPO1–4) are ligands for three transmembrane receptors, leucine-rich repeat-containing G-protein coupled receptors 4–6 (LGR4–6) [2, 3]. As shown schematically in Figure 1, RSPO binding to LGR4–6 enhances Wnt signaling by inhibiting negative regulators of the Wnt pathway. Among the genes upregulated by β-catenin are *ZNRF3* and *RNF43*, functionally homologous cell-surface transmembrane proteins that ubiquitinate FZD and LRP5/6 receptors, leading to their endocytosis and degradation by the lysosome. This has the effect of dampening Wnt signaling as part of a negative feedback loop [4, 5]. RSPOs bind simultaneously to the extracellular domains of LGR4–6 and ZNRF3/RNF43, forming a ternary complex that is cleared from the membrane. This results in prolonged cell-surface residence time of FZD and LRP5/6 and enhancement of Wnt signaling [6, 7]. Hao *et al.* elegantly demonstrated that the proximity of ZNRF3/RNF43 to LGR4–6 is crucial for membrane clearance by engineering a dimerization interface between ZNRF3 and LGR4, which resulted in loss of ZNRF3 from the membrane upon treatment with the dimerizer [8]. RSPO proteins therefore serve as matchmakers to join LGR4–6 with ZNRF3/RNF43. The exact mechanism of membrane clearance is yet to be determined but depends on the intracellular RING domain of ZNRF3/RNF43 [8].

LGR5 and LGR6 have gained prominence because they are among the few known stem cell-specific receptors. Studies in mice have shown that LGR5 marks epithelial stem cells in the intestine, hair follicle, kidney, mammary gland, colon, and ovary [9–13]; LGR6 marks stem cells in the epidermis, mammary gland, nail, and fallopian tube [14–17]. The presence of LGR5 and LGR6 on stem cells is consistent with the elevated Wnt signaling in these cells. Importantly, LGR5 and LGR6, and their ligands RSPO1–4, are upregulated in several cancers, lending support to the idea that elevated Wnt signaling may be a driver of tumor formation [18–21]. The third RSPO receptor, LGR4, is expressed more broadly and is not a marker of stem cells per se [22, 23], although it has important roles in intestinal morphogenesis [24], hair, prostate, and mammary gland development [25–27], and tumor formation and metastasis [28–31]. This review will focus on the regulation of epithelial stem cells by LGR5 and LGR6 and the contribution of this signaling pathway to tumor formation and chemoresistance. Particular emphasis will be given to stem cells of the ovary and fallopian tube epithelia, two tissues that are implicated in ovarian cancer formation.

### Organization of the intestinal stem cell niche

In the mouse intestinal epithelium, stem cells are located at the base of the crypts and are intercalated with differentiated Paneth cells. The stem cells are marked by their expression of FZD and LGR5 receptors, while Paneth cells express CD24 and secrete EGF, TGFα, Wnt3 and the Notch ligand Dll4 [32]. Paneth cells are an important source of ligands for the receptors that support stem cell function. As the stem cells divide, they give rise to daughters that either remain as stem cells in the niche or become transit-amplifying cells that differentiate along several lines. Some transit-amplifying cells become Paneth cells and remain at the base of the crypt, while others proliferate and migrate up the side wall of the crypts in a conveyor belt-like fashion as they become absorptive epithelium cells, goblet cells, and enteroendocrine cells. After service on the gut surface for several days, they undergo apoptosis and are lost into the lumen [33, 34].

There are 19 human Wnt ligands and 10 FZD receptors, making it a considerable challenge to identify the combinations that are important in stem cell function. In contrast, the lower number of RSPOs and LGRs has allowed characterization of their expression and roles in stem cells. In the mouse intestinal crypt, LGR5 marks a cell at the crypt base that can generate all of the cell types found on the villus [35]. The expression level of the RSPOs is crucial for maintaining the proper number of stem cells and limiting tissue growth [36–38]. Overexpression of *RSPO1* or *RSPO2* in mice resulted in crypt hyperplasia and increased expression of Wnt target genes [36, 38]. Similarly, *RSPO3* overexpression caused intestinal hyperplasia and the formation of adenomas that sometimes progressed to adenocarcinomas [37]. These studies show that limiting the amount of RSPO1–3 is important in preventing aberrant cell growth, but do not allow a clear determination of the native RSPOs that regulate stem cell function in the crypt. RSPOs can substitute for each other in tissue culture experiments [7], and it is plausible that high-level expression of any isoform, results in increased Wnt activity and hyperplasia.

Surprisingly, neither RSPO1 nor LGR5 are necessary for the wellbeing of the murine gut epithelium under steady-state conditions, although both appear to play a role in recovery from injury. Knockout of *RSPO1* in mice caused aberrant ovarian masculinization, but no reported defects in intestinal development [39–41]. Humans with hereditary loss of *RSPO1* have the skin-thickening condition palmoplantar hyperkeratosis, increased susceptibility to squamous cell carcinoma, and XX sex reversal, but no reported intestinal abnormalities [42, 43]. Conditional knockout of *LGR5* in adult mice similarly caused no apparent defects in intestinal structure, possibly due to the presence of LGR4 in the stem cell population [3]. This lack of intestinal defects in *RSPO1*- and *LGR5*-null mice likely reflects the fact that Wnt signaling still occurs in the absence of these genes, and this is apparently sufficient for normal tissue development and maintenance. Following injury, however, Wnt signaling increases [44], and RSPO1 and LGR5 become important in recovery of the epithelium. When intestinal cells were killed by the chemotherapeutic agent 5-fluorouracil, overexpression of recombinant *RSPO1* ameliorated cell loss [36]. Mice overexpressing *RSPO1* also had improved structural and functional regeneration of the intestine, including recovery of stem cell populations, following abdominal irradiation [45]. When LGR5-expressing cells were depleted in the crypt prior to radiation treatment, the crypt failed to regenerate [46]. These results demonstrate that the increase in Wnt activity that facilitates recovery from injury occurs at least in part through RSPO signaling to LGR receptors.

A central question in Wnt stem cell biology is how RSPO and Wnt ligands cooperatively control stem cell self-renewal and differentiation. An important recent paper from Yan *et al.* helped define the roles of each ligand in mouse intestinal crypt stem cells [38]. RSPOs regulate the size of the stem cell pool, as overexpression of *RSPO1* or *RSPO2* resulted in the expansion of LGR5^+^ stem cells. Reduction in RSPO signaling through the expression of the ectodomains of RSPO-binding proteins caused a marked loss of stem cells and an increase in differentiated progeny. In contrast, expression of a Wnt ligand analogue did not regulate stem cell number, but was required for stem cell expansion in response to RSPO. Inhibition of Wnt ligands resulted in lower expression of *LGR5*, *ZNRF3*, and *RNF43*, demonstrating that Wnt ligands prime stem cells for RSPO signaling through the expression of its receptors [38]. These results are consistent with the finding that RSPO1 and LGR5 are expendable for normal function of the intestine when stem cell populations are static, but are important for stem cell expansion following injury. In regard to cancer, stem cells are relatively resistant to chemotherapeutic agents [47, 48], and increased levels of LGR5 correlate with chemoresistance [49–51], suggesting that LGR signaling may promote the recovery of a cancer stem cell population following chemotherapy.

### LGRs and Wnt signaling in skin stem cells

The stem cell niches of other epithelia differ in their morphology but appear to have a similar structural organization to the intestine, in which stem cells are supported by factors released from cells in their immediate environment [52]. In the adult mouse skin, stem cell populations that regulate the interfollicular epidermis (IFE) and hair follicle (HF) have been identified in the basal layer of the IFE, the bulge and hair germ regions of the HF, and the sebaceous gland [53]. The individual populations of stem cells are multipotent and capable of regenerating different structures in the skin following injury [54]. Among the LGR proteins, the mostly likely contributors to Wnt signaling in the HF are LGR4 and LGR5, the latter of which was found in an actively cycling population in the hair germ that was capable of reconstituting hair follicles when grafted onto nude mice [11].

While the population of LGR5^+^ cells were found to control HF growth, they did not contribute to the growth or self-renewal of the sebaceous gland or IFE [11]. Instead, these tissues express LGR6, demonstrating heterogeneity in the type of LGR receptor on stem cells within an organ [14]. Although initially thought to function independently of Wnt [14], mouse IFE stem cells in the basal epidermis were shown to express the canonical Wnt marker Axin2, and inhibition of β-catenin reduced proliferation [55]. Thus, LGR6 in IFE cells is likely functioning in a canonical manner to regulate Wnt activity in the stem cell population. Interestingly, the IFE stem cells function as their own niche by expressing Wnt ligands, an example of autocrine signaling within the stem cell environment [55].

The expression of LGR5 and LGR6 in skin stem cells and the role of Wnt signaling suggests the presence of RSPOs as ligands, although few studies have examined this. The first evidence for RSPO function in skin was through mapping of a familial propensity to palmoplantar hyperkeratosis and squamous cell carcinoma to loss-of-function mutations in *RSPO1* [42]. Later molecular characterization in mice showed that all four isoforms of RSPO were expressed in the skin, and that overexpression of *RSPO1* activated the Wnt pathway, including increased expression of *LGR5*, and resulted in defects in HF growth [56]. RSPO2 also is linked to skin and hair development. Keloids, raised scars caused by overgrowth of granulation tissue, contain keratinocytes that express elevated levels of *RSPO2* [57]. Studies on the genetic basis of coat variation in domestic dogs have found linkage between alleles of *RSPO2* and fur type [58, 59]. These findings indicate a role for RSPO/LGR activity in skin and skin appendage development, and further demonstrate that tight regulation of Wnt signaling is important for maintaining the normal function of stem cells.

### Wnt signaling in ovarian and fallopian tube stem cells and ovarian cancer

Ovarian cancer is the fifth-leading cause of cancer deaths among women in the United States. The most common and malignant type of ovarian cancer is high grade serous ovarian carcinoma (HGSOC). The tissue origins of HGSOC are controversial, and have been attributed to two juxtaposed epithelia: the ovarian surface epithelium (OSE) that encapsulates the ovary, and the fallopian tube epithelium (FTE) at the distal end of the fallopian tube in contact with the ovary [60]. The OSE is comprised of a single type of cuboidal cell, whereas the FTE is a mix of ciliated and secretory cells. Cells are shed from the FTE into the peritoneal cavity and these cells can embed onto the ovarian surface. Such a migration of transformed cells from the FTE to the ovary, a site more supportive of proliferation, has been proposed to be the first step in the formation of HGSOC [60].

In contrast to the intestine and skin, which have been extensively studied as models of epithelial stem cell biology, considerably less is known about the stem cells present in the OSE and FTE. This is due to the difficulty of isolating and culturing these cells to allow molecular characterization [61]. Studies in mice and humans have shown that both populations of stem cells are regulated by Wnt signaling [15, 62]. Furthermore, Wnt signaling is upregulated in ovarian tumors [63], and is an important contributor to the acquired resistance of ovarian cancers to chemotherapeutics, a key factor in the high mortality of ovarian cancer [64–66]. Given the significance of RSPO/LGR activity in increasing WNT signaling, it is likely that RSPO and LGR proteins also factors into tumor formation and chemoresistance in the OSE and FTE.

### LGR5 and RSPO1 function in OSE stem cells

LGR5 is expressed in stem cells in the mouse OSE starting in embryogenesis and continuing into adulthood [67, 68]. In the adult mouse, LGR5 is found in cell populations that co-express CD33, ALDH, and Ki67 [12, 67]. The OSE ruptures to allow release of the oocyte during ovulation, and LGR5 was expressed in regions where rupture occurs, consistent with a role for stem cells in tissue repair [67]. LGR5-expressing OSE stem cells exhibited increased tumorigenicity in a *TP53*- and RB1-deficient background, suggesting that these cells may mediate tumor formation [12].

The expression pattern of RSPO1 in the ovary and its stimulation of cell growth indicates a likely role in stem cell regulation. It is expressed broadly in the developing mouse ovary during embryogenesis, where it is essential for female gonad development [69]. After birth it becomes limited to a subset of cells that includes the OSE [70]. Given that LGR5 is also expressed in this cell population [67], it appears that the niche for OSE stem cells is either adjacent differentiated cells, similar to the Paneth cells intercalated among stem cells in the intestine, or the LGR5^+^ cells themselves through an autocrine signaling mechanism. Increasing levels of RSPO1 in the mouse ovary by expression from the *Sf1* promoter produced granulosa cell tumors [70]. This is analogous to the findings that overexpression of RSPOs in the intestine causes hyperplasia [36–38], and support the finding that RSPO1 is an oncogene when expressed at elevated levels [18].

### RSPO1 and LGR6 function in fallopian tube stem cells

**Figure 2:**
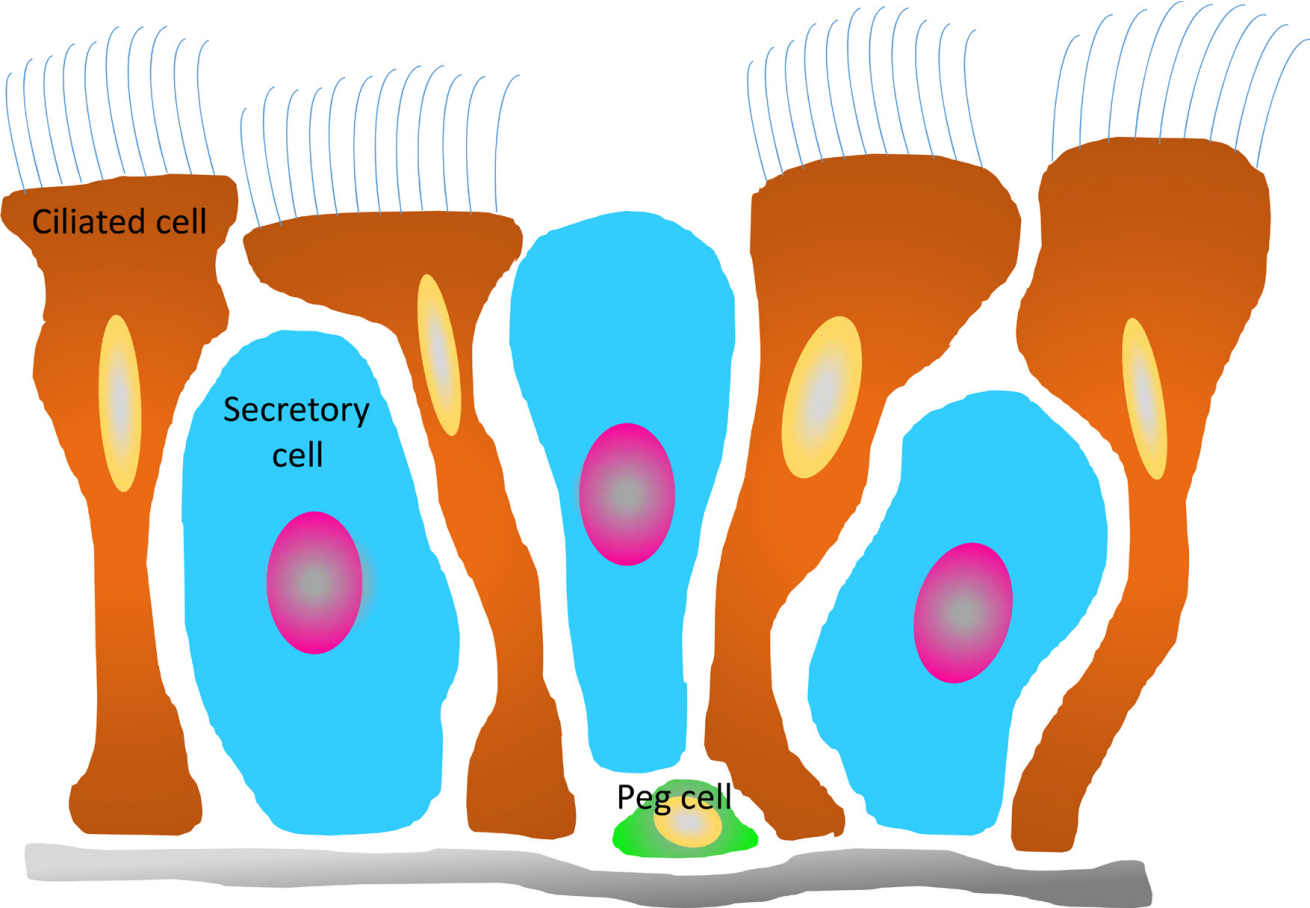
Schematic diagram of cellular organization of the fallopian tube epithelium The PEG cells (green) have been identified as precursors of both the secretory (blue) and ciliated (brown) cells, but which cell type serves as the primary source of RSPO1/2 is unknown.

The predominant cell types in the FTE are secretory and ciliated cells, comprising 90% of the population. There is a third type of cell, the peg cell, which is a candidate for the role of FTE stem cell. Peg cells are narrow, undifferentiated cells concentrated in the fimbria that are capable of self-renewal and clonal growth (Figure 2). Isolated human peg cells can grow into spheroids that produce secretory and ciliated cells, indicating that they are multipotent [71]. Peg cells can be distinguished from other cells in the FTE by their expression of CD44 and cytokeratin 5 (KRT5). Importantly, examination of clinical samples from patients with invasive serous carcinomas were positive for CD44 and KRT5, indicating that peg cells may be an important FTE cell population in the formation of ovarian tumors [71].

Analysis of cultured fallopian tube stem cells points to a role for RSPO1 signaling. Kessler *et al.* [15] isolated Ki67^+^ fallopian tube epithelial cells from patient samples and grew them into organoids *in vitro* using a cocktail of growth factors that included Wnt3A, EGF, and FGF. The organoids contained both ciliated and secretory cells, suggesting recapitulation of the FTE. Addition of RSPO1 to the growth medium increased Wnt signaling, the number of organoids, and organoid size [15]. In contrast to the OSE, which expresses *LGR5* [12], microarray and whole-genome sequence analysis of FTE organoids revealed elevated expression of *LGR6* but not *LGR5* [15, 72]. LGR6 was found to reside in a multipotent cell population in organoids that expressed the proliferation marker Ki67 [15, 72]. When stem cells in organoids were induced to differentiate by blocking Notch signaling with the γ-secretase inhibitor DBZ, *LGR6* expression was reduced, suggesting that loss of stem cell self-renewal involves downregulation of *LGR6* expression [15].

### Expression of *LGR5*, *LGR6*, *RSPO1*, and *RSPO2* in high grade serous ovarian cancer

**Figure 3:**
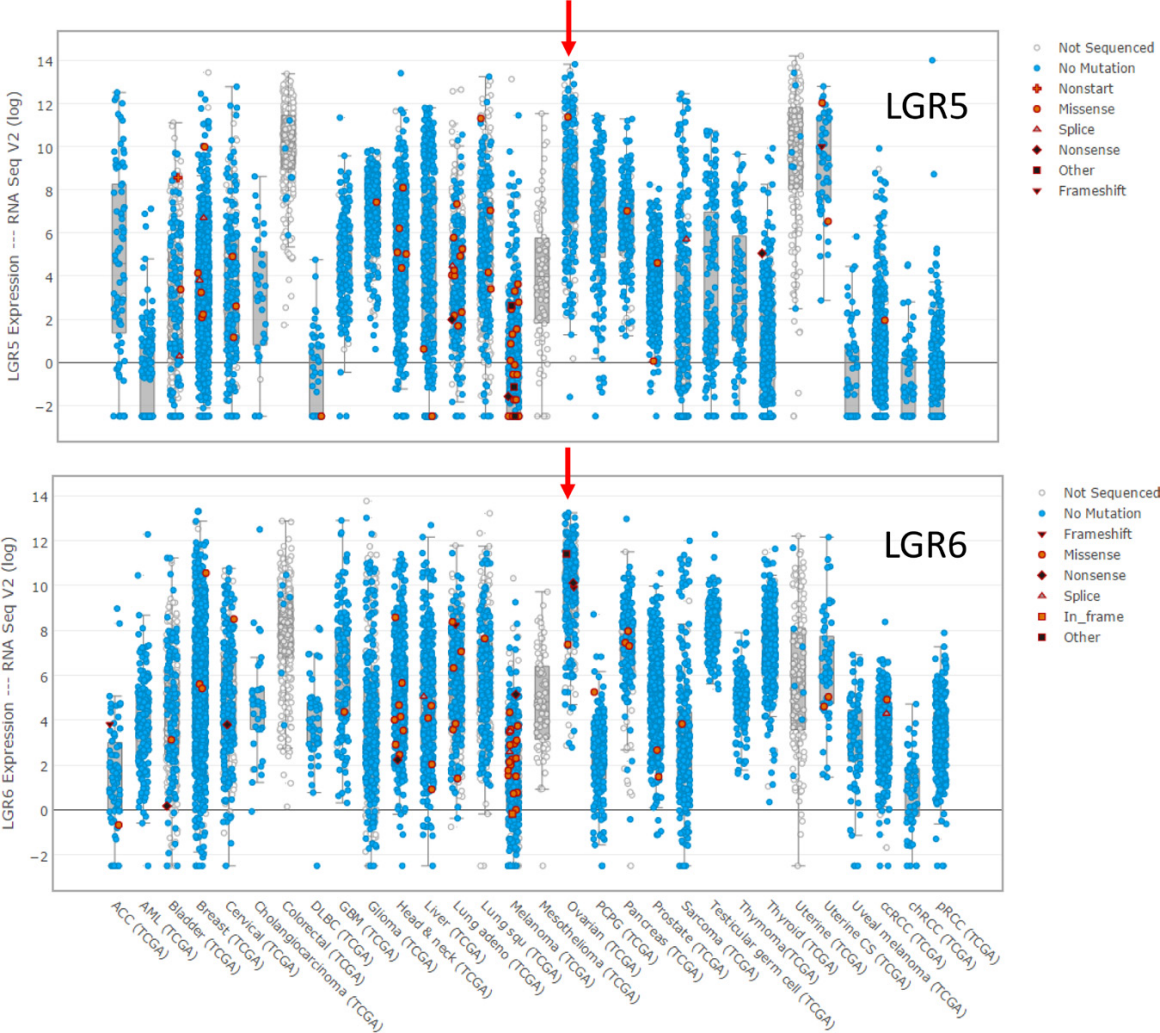
Expression of *LGR5* and *LGR6* at the mRNA level in the tumors in the TCGA database for which expression data is reported

Despite the evidence that LGR5 is the major RSPO receptor in OSE stem cells and LGR6 is the primary receptor in FTE stem cells, data from The Cancer Genome Atlas (TCGA) suggest that both may be important in HGSOC. Both genes are amplified at a low frequency in ovarian cancers (*LGR5* 4%; *LGR6* 8%), but HGSOC is characterized by copy number variations at many loci, and neither gene stands out in this regard. No mutations were found in *LGR5*, and very few mutations were found in *LGR6*. More interesting is the observation that both genes are highly expressed in HGSOC compared to other tumors in the TCGA database (Figure 3). HGSOC, uterine, and colorectal cancers have the highest levels of *LGR5* mRNA, and HGSOC has the highest level of *LGR6* expression.

**Figure 4:**
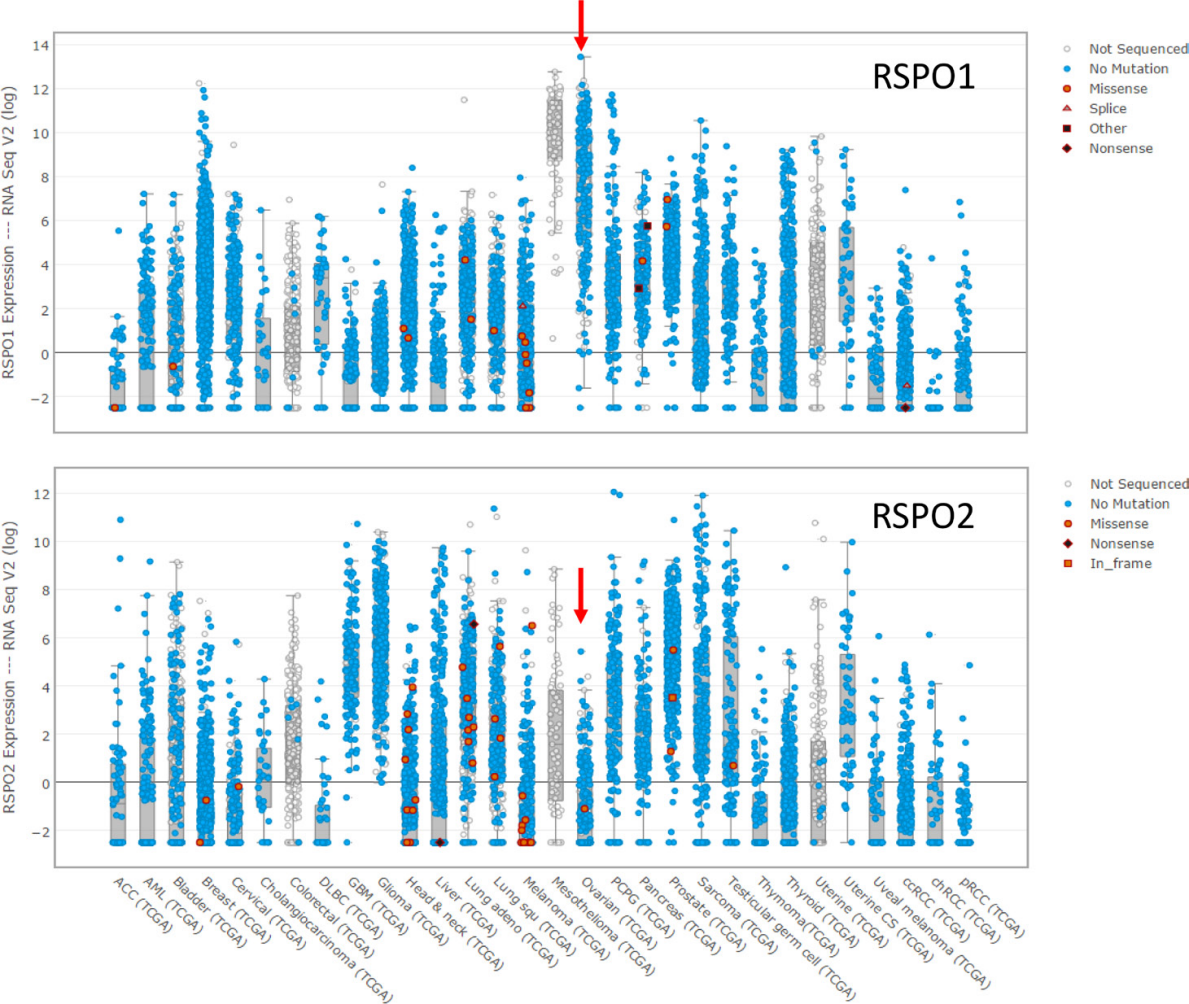
Expression of *RSPO1* and *RSPO2* at the mRNA level in the tumors in the TCGA database for which expression data is reported

As with *LGR5* and *LGR6*, neither *RSPO1* nor *RSPO2* is mutated, although both exhibit low levels of amplification in HGSOC (*RSPO1* 9%; *RSPO2* 24%). The differences in expression at the mRNA level, however, are quite pronounced (Figure 4). HGSOC has the highest level of *RSPO1* mRNA expression among all epithelial carcinomas, and one of the lowest levels of *RSPO2* expression. Importantly, a genome-wide association study identified polymorphisms in *RSPO1* as a risk factor for ovarian cancer, and specifically for tumors of serous histology [73]. There was no significant correlation between the mRNA expression of any of the four *RSPOs* and *LGR5*; only *RSPO1* is significantly correlated with the expression of LGR6 in HGSOC (Pearson 0.478). Overall, the currently available data does not permit an assessment of the extent to which these ligands and receptors drive the high level of Wnt signaling that is found in HGSOC [63, 66]. Single cell studies are required to determine whether a given tumor cell expresses one or both LGR receptors, and whether tumor cells or a stromal cell type is the source of RPSO ligands. How expression of any of these molecules in tumor cells is linked to the magnitude of Wnt signaling remains to be addressed.

### Summary and conclusions

Despite anatomical differences, epithelial stem cells in diverse tissues appear to be organized in similar ways. Differentiated niche cells are located in close proximity to the stem cells and secrete Wnt and RSPO ligands that regulate the canonical Wnt signaling pathway. The stem cells, in turn, express Wnt receptors FZD and LRP5/6, and RSPO receptors LGR4–6. The RSPO/LGR interaction serves to fine tune Wnt signaling. Although the RSPOs and LGR5–6 are dispensable in many cases for the steady-state functioning of tissues, they plays a role in recovery from injury, a process that requires elevated Wnt signaling. This suggests that RSPO/LGR activity, which is uniquely a vertebrate adaptation [74], may have evolved as a way to signal from the epithelium to resident stem cells to increase the rate of tissue growth. This advantageous aspect of RSPO/LGR activity has a parallel dark side: the risk of cancer if Wnt signaling is aberrantly elevated. Given their roles as Wnt signaling modulators and potential oncogenes, a key unresolved question is how RSPO and LGR expression activity is regulated. Recent work showing that Wnt signaling primes stem cells to receive RSPO ligands by increasing expression of its receptors provides an important insight into the cooperativity of Wnt and RSPO ligands in regulation of stem cells populations [38]. The study also found that RSPO ligands, not Wnts, were the key determinant of stem cell number [38]. The centrality of RSPO/LGR signaling in stem cells highlights the importance of further understanding the regulation of this pathway at the cellular level.

The identification of LGR5 and LGR6 as stem cell markers has presented a way to identify and isolate stem cells in a range of tissues. It also points toward a therapeutic strategy based on targeting these receptors to reduce cell proliferation. It may be possible to enhance selectivity by developing receptor antagonists that prevent the increase in stem cell number mediated by RSPOs without disabling the basal Wnt signaling required to keep normal stem cells alive. Such therapies would have potential benefits in the treatment of ovarian cancer, which is often diagnosed at a late stage and becomes resistant to chemotherapy [75]. To the extent that Wnt signaling reduces sensitivity to the platinum drugs and PARP inhibitors, inhibiting LGR/RSPO activity could make chemotherapeutics more effective in the management of HGSOC. The identification of the LGR/RSPO axis as a Wnt enhancer system in stem cells opens avenues to better isolate, characterize, and target a population of cells that is important to tumor formation and chemoresistance.
